# Aurora-A kinase oncogenic signaling mediates TGF-β-induced triple-negative breast cancer plasticity and chemoresistance

**DOI:** 10.1038/s41388-021-01711-x

**Published:** 2021-03-05

**Authors:** Mohammad Jalalirad, Tufia C. Haddad, Jeffrey L. Salisbury, Derek Radisky, Minzhi Zhang, Mark Schroeder, Ann Tuma, Eduard Leof, Jodi M. Carter, Amy C. Degnim, Judy C. Boughey, Jann Sarkaria, Jia Yu, Liewei Wang, Minetta C. Liu, Luca Zammataro, Lorenzo Malatino, Evanthia Galanis, James N. Ingle, Matthew P. Goetz, Antonino B. D’Assoro

**Affiliations:** 1 Department of Oncology, Mayo Clinic College of Medicine, Rochester, MN USA; 2 Department of Biochemistry and Molecular Biology, Mayo Clinic College of Medicine, Rochester, MN USA; 3 Department of Radiation Oncology, Mayo Clinic College of Medicine, Rochester, MN USA; 4 Department of Laboratory Medicine and Pathology, Mayo Clinic College of Medicine, Rochester, MN USA; 5 Department of Surgery, Mayo Clinic College of Medicine, Rochester, MN USA; 6 Department of Oncology, Yale University, New Heaven, CT USA; 7 Department of Clinical and Experimental Medicine, University of Catania, Catania, Italy; 8 Department of Molecular Medicine, Mayo Clinic College of Medicine, Rochester, MN USA

**Keywords:** Breast cancer, Hormone receptors

## Abstract

Triple-negative breast cancer (TNBCs) account for 15–20% of all breast cancers and represent the most aggressive subtype of this malignancy. Early tumor relapse and progression are linked to the enrichment of a sub-fraction of cancer cells, termed breast tumor-initiating cells (BTICs), that undergo epithelial to mesenchymal transition (EMT) and typically exhibit a basal-like CD44^high^/CD24^low^ and/or ALDH1^high^ phenotype with critical cancer stem-like features such as high self-renewal capacity and intrinsic (de novo) resistance to standard of care chemotherapy. One of the major mechanisms responsible for the intrinsic drug resistance of BTICs is their high ALDH1 activity leading to inhibition of chemotherapy-induced apoptosis. In this study, we demonstrated that aurora-A kinase (AURKA) is required to mediate TGF-β-induced expression of the SNAI1 gene, enrichment of ALDH1^high^ BTICs, self-renewal capacity, and chemoresistance in TNBC experimental models. Significantly, the combination of docetaxel (DTX) with dual TGF-β and AURKA pharmacologic targeting impaired tumor relapse and the emergence of distant metastasis. We also showed in unique chemoresistant TNBC cells isolated from patient-derived TNBC brain metastasis that dual TGF-β and AURKA pharmacologic targeting reversed cancer plasticity and enhanced the sensitivity of TNBC cells to DTX-based-chemotherapy. Taken together, these findings reveal for the first time the critical role of AURKA oncogenic signaling in mediating TGF-β-induced TNBC plasticity, chemoresistance, and tumor progression.

## Introduction

Triple-negative breast cancer (TNBC) accounts for 15–20% of all breast cancers [1]. In non-metastatic TNBC treated with neoadjuvant chemotherapy (NAC), women with residual disease after NAC have high rates of distant tumor relapse occurring within 1–3 years after treatment, and these recurrences are associated with a high incidence of mortality [2]. One of the major limitations in eradicating TNBC metastasis is the enrichment of breast tumor-initiating cells (BTICs) that give rise to tumor cell heterogeneity, self-renewal capacity, and intrinsic (de novo) resistance to chemotherapeutic agents [3–5]. Because limited FDA-approved targeted therapies are currently available for TNBCs [6], a better characterization of the cross-talk between key oncogenic pathways responsible for cancer plasticity and enrichment of BTICs will be essential to develop novel targeted-therapies to enhance chemosensitivity and inhibit TNBC progression.

A well-characterized oncogenic pathway that plays a central role in TNBC progression is TGF-β signaling [7, 8]. TGF-β signal transduction starts with TGF-β1 (the major isoform) binding to a complex of specific serine/threonine kinase receptors (TβRI and TβRII). This interaction leads to phosphorylation/activation of regulatory SMAD2/SMAD3 transcription factors that in turn bind the common mediator SMAD4 in the nucleus and regulate target gene expression [9]. In normal epithelial cells and early-stage luminal breast tumors, TGF-β/SMAD signaling induces cell cycle arrest and activation of apoptosis [10, 11]. On the contrary, in TNBC, TGF-β signaling drives tumor-promoting responses such as chemoresistance and early emergence of metastasis [12, 13]. TGF-β signaling promotes an invasive phenotype through activation of epithelial to mesenchymal transition (EMT) reprogramming leading to cancer cell plasticity and tumor progression [14]. Changes during EMT drive the transition from a polarized epithelial phenotype to an elongated fibroblastoid-like phenotype that typifies the morphology of most TNBCs. Mechanistically, TGF-β signaling promotes EMT and the resulting cancer plasticity by inducing the expression of EMT-activating transcription factors (ATFs) [15]. EMT-ATFs act as molecular switches because they down-regulate epithelial proteins (E-cadherin and claudin) responsible for cell adhesion and up-regulate mesenchymal proteins (N-cadherin, vimentin, and fibronectin) involved in cell motility and invasion. One of the major EMT-ATFs is SNAI1 that plays a key role in promoting chemoresistance, metastasis, and tumor progression [16, 17]. Likewise, breast cancer cells that show EMT-mediated cancer plasticity also acquire a CD44^high^/CD24^low^ and/or ALDH^high^ cancer stem-like phenotype characterized by an increased capacity for tumor self-renewal that will drive intrinsic drug resistance and high metastatic proclivity [18]. High ALDH1 activity is a better universal functional marker predictive of enrichment of chemoresistant BTICs as compared to CD44^high^/CD24^low^ breast cancer stem-like cell markers that cannot be ubiquitously used to identify BTICs in all breast cancer subtypes [19]. High ALDH1 activity induces intrinsic drug resistance through ALDH1-mediated detoxification of toxic aldehyde intermediates produced in cancer cells following treatment with standard chemotherapy [20].

A key oncoprotein that also plays an important role in breast cancer progression is the Aurora-A serine/threonine mitotic kinase (AURKA). AURKA localizes to centrosomes and mitotic spindles of dividing cells [21] and modulates centrosome duplication and spindle formation for appropriate chromosome segregation during mitosis ensuring the maintenance of chromosomal stability [22, 23]. When AURKA is over-expressed in human breast tumors, it is commonly associated with an invasive basal-like phenotype and poor prognosis [24–26]. Several studies have demonstrated that aberrant AURKA activity plays a key role in breast cancer progression through the development of centrosome amplification and chromosomal instability (CIN) [27–30]. AURKA-induced CIN represents one of its most established oncogenic functions because the emergence of CIN confers tumor heterogeneity, drug resistance, and poor outcome [31, 32]. AURKA also plays a central role in the progression of solid tumors through activation of EMT and tumor stemness [33–35]. Significantly, we have demonstrated the central role of AURKA in promoting drug resistance and breast cancer progression through activation of EMT reprogramming and the enrichment of highly invasive BTICs [36, 37]. Nevertheless, the molecular mechanisms by which aberrant AURKA activity induces cancer cell plasticity, chemoresistance, and progression in TNBCs are largely unknown. Because of their role in EMT-mediated cancer cell plasticity, drug resistance and ultimately poor clinical outcome, TGF-β and AURKA oncogenic pathways represent attractive druggable targets, and several small molecule inhibitors of TGF-β and AURKA activity are under clinical investigation [38–42].

In this study, we demonstrated for the first time that AURKA is required to mediate TGF-β-induced expression of a SNAI1 invasive regulatory network, enrichment of ALDH1^high^ BTICs, self-renewal capacity, and chemoresistance in TNBC experimental models. The combination of docetaxel (DTX) with dual TGF-β (galunisertib) and AURKA (alisertib) pharmacologic targeting impaired tumor relapse and the emergence of distant metastasis. We also showed in unique TNBC cells isolated from patient-derived TNBC brain metastasis that dual TGF-β and AURKA pharmacologic targeting reversed cancer plasticity and impaired tumor stemness. Significantly, the combination of galunisertib and alisertib also enhanced the sensitivity of TNBC cells to DTX-based-chemotherapy. Taken together, these findings reveal a novel non-mitotic role of AURKA oncogenic signaling in mediating TGF-β-induced TNBC plasticity and chemoresistance, providing a compelling preclinical rationale for the design of innovative therapeutic strategies that will selectively target the TGF-β/AURKA oncogenic axis to impair chemoresistance and TNBC progression.

## Results

### Aberrant AURKA expression is linked to the shorter overall survival of TNBC patients

**Fig. 1:**
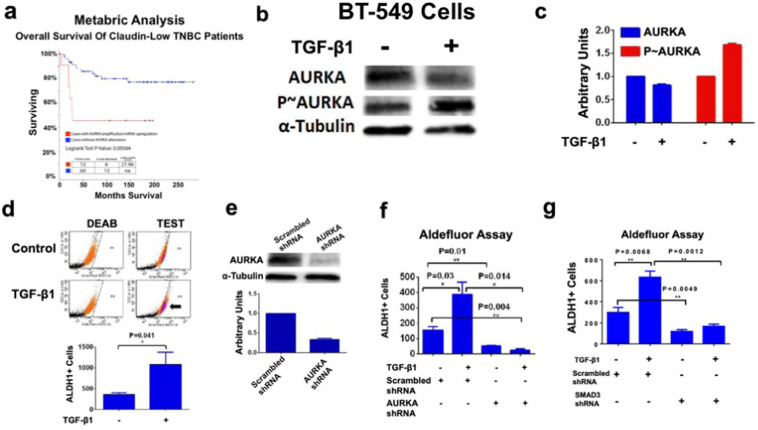
AURKA expression is essential to mediate TGF-β-Induced ALDH activity. **a** The Claudin low (CL)-TNBC subgroup (72 cases) showed an average age at diagnosis of 61.9 years, while the overall survival average was 126.5 months. The median months' survival calculated for the whole follow-up was 27.96 months. In total, 12/72 cases (17%) exhibited AURKA alterations (mRNA up-regulation and/or copy number variations), while none of 72 patients harbored deletions or down-regulations of AURKA. Survival analysis showed that aberrant AURKA expression was significantly associated with reduced patient overall survival (*p*-value = 0.00584). **b** Immunoblot assay showing expression of total and phosphorylated AURKA in BT-549 cells before and after treatment with TGF-β1 (10 ng/ml) for 48 h. **c** Densitometry analysis showing the fold change of total and phosphorylated AURKA protein levels in BT-549 TNBC cells normalized to α-Tubulin. Experiments were performed in triplicate. **d** BT-549 cells were treated with TGF-β1 (10 ng/ml) for 48 h. After 48 h incubation, ALDH1 activity was detected with Aldefluor kit and measured by FACS analysis on 10,000 events. ALDH1 inhibitor DEAB was used as control. Graph showing the average of ALDH1^High^ cells from three independent experiments (±s.d.). **e** Immunoblot assay showing expression of total AURKA in BT-549 cells infected with scrambled and AURKA lenti-shRNAs for 48 h. Densitometry analysis showing the fold change of total AURKA protein levels in BT-549 TNBC cells normalized to α-Tubulin. **f** BT-549 cells were treated with 10 ng/ml TGF-β1 and lenti-shRNAs. After 48 h incubation, ALDH1 activity was detected with Aldefluor kit and measured by FACS analysis on 10,000 events. ALDH1 inhibitor DEAB was used as the control for each sample. Graph showing the average of ALDH1^High^ cells from three independent experiments (±s.d.). **g** BT-549 cells were treated with 10 ng/ml TGF-β1, scrambled lenti-shRNAs, and lenti-shRNAs targeting SMAD3. After 48 h incubation, ALDH1 activity was detected with Aldefluor kit and measured by FACS analysis on 10,000 events. ALDH1 inhibitor DEAB was used as the control for each sample. Graph showing the average of ALDH1^High^ cells from three independent experiments (±s.d.).

In order to define the putative association between increased AURKA expression and shorter survival of TNBC patients, we have analyzed genome sequencing and mRNA-seq data of specimens from the TCGA and breast cancer METABRIC studies [43]. For this analysis, we have selected the highly aggressive Claudin low-TNBC (CL-TNBC) subgroup because it is characterized by an enrichment of genes linked to TGF-β signaling pathway [44]. The CL-TNBC subgroup (72 cases) showed an average age at diagnosis of 61.9 years with an overall survival average was 126.5 months. The median months survival calculated for the whole follow-up was 27.96 months in 12/72 cases (17%) that exhibited AURKA alterations (mRNA up-regulation and/or copy number variations). None of the 72 patients harbored deletions or down-regulations of AURKA. Aberrant AURKA expression was significantly associated with reduced overall survival (Fig. 1a), suggesting that AURKA oncogenic signaling promotes TNBC progression and poor outcome. We also investigated the correlation between increased AURKA expression and shorter survival of patients with luminal ER+, PR+, HER-2- and basal-like ER-, PR-,HER-2+ breast cancer subtypes. However, aberrant AURKA expression was not significantly associated with reduced overall survival in these breast cancer subtypes (Supplementary Fig. 1).

### AURKA expression is necessary to mediate TGF-β-induced enrichment Of ALDH1^High^ TNBC cells

We then aimed to define the mechanistic linkage between TGF-β and AURKA oncogenic pathways in established TNBC cell lines. To investigate the role of TGF-β signaling in inducing AURKA *Threonine-288* phosphorylation that in turn mediates AURKA-induced activation of down-stream targets [36, 37], BT-549 TNBC cells (mesenchymal-like subtype) were treated with TGF-β1. TGF-β1 increased the levels of phosphorylated AURKA that was not associated with an increase of total AURKA used as control (Fig. 1b, c). An Aldefluor assay was performed in BT-549 cells to investigate whether TGF-β1-induced AURKA phosphorylation was linked to the enrichment of cells with high ALDH1 activity, which defines a BTICs phenotype [45]. As expected, treatment of BT-549 cells with TGF-β1 induced an enrichment of ALDH1^high^ cells (Fig. 1d).

Next, to establish the role of AURKA expression in mediating TGF-β-induced ALDH1 activity, BT-549 cells were infected with scrambled lenti-shRNAs or lenti-shRNAs targeting AURKA mRNA (Fig. 1e). BT-549 cells infected with scrambled lenti-shRNAs or lenti-AURKA shRNAs were treated with TGF-β1 and the enrichment of ALDH1^high^ cells was determined by aldefluor assay. Significantly, AURKA genetic targeting inhibited TGF-β-induced ALDH1 activity in BT-549 cells (Fig. 1f and Supplementary Fig. 2a). The role of AURKA in mediating TGF-β-Induced enrichment of ALDH^high^ cells was also corroborated in MDA-MB 231 TNBC cells that show a claudin-low mesenchymal stem-like phenotype (Supplementary Fig. 2b, c). AURKA expression was also required to mediate TGF-β-Induced EMT (Supplementary Fig. 3a). To establish whether TGF-β-induced AURKA phosphorylation and ALDH1 activity were also linked to CIN, we analyzed the grade of centrosome amplification (CA), a well-established marker of CIN. Treatment of TNBC cells with TGF-β1 for 48 h did not induce an increase of CA (Supplementary Fig. 3b). To define the extent to which SMAD3 targeting (Supplementary Fig. 3c) also reduced the enrichment of ALDH1^High^ cells, BT-549 cells infected with scrambled lenti-shRNAs or lenti-SMAD3 shRNAs were treated with TGF-β1 and the enrichment of ALDH1^high^ cells was determined by aldefluor assay. SMAD3 genetic targeting inhibited TGF-β-induced ALDH1 activity in BT-549 cells (Fig. 1g and Supplementary Fig. 3d). Significantly, AURKA pharmacologic targeting did not reduce SMAD3 phosphorylation (Supplementary Fig. 3e), indicating that TGF-β induced the enrichment of ALDH1^high^ TNBC cells through distinct canonical SMAD3 and non-canonical AURKA signaling pathways.

### High ALDH1 activity is required to mediate AURKA-induced self-renewal capacity

**Fig. 2:**
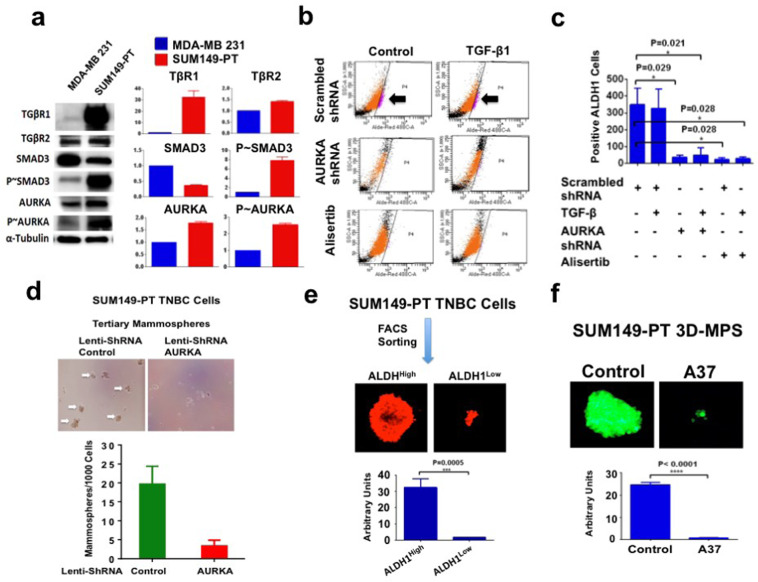
ALDH1 activity mediates AURKA-induced self-renewal capacity. **a** Immunoblot assay showing expression of TGβR1, TGβR2, SMAD3 (total and phosphorylated), and AURKA (total and phosphorylated) proteins in MDA-MB 231 and SUM149-PT cells. Densitometry analysis showing the fold change of protein levels in MDA-MB 231 and SUM149-PT cells normalized to α-Tubulin. **b** SUM149-PT cells were treated with 10 ng/ml TGF-β1, lenti-shRNAs, or alisertib. After 48 h incubation, ALDH1 activity was detected with Aldefluor kit and measured by FACS analysis on 10,000 events. ALDH1 inhibitor DEAB was used as the control for each sample. **c** Graph showing the average of ALDH1^high^ cells from three independent experiments (±s.d.). **d** SUM149-PT cells were cultured under non-adherent conditions for 24 days (three serial passages) to form tertiary mammospheres (MPS). In total, 1000 SUM149-PT cells derived from tertiary MPS were then infected with scrambled or AURKA lenti-ShRNAs and were incubated for 8 days to monitor MPS growth. Graph showing the average of ALDH1^High^ cells from three independent experiments (±s.d.). **e** FACS sorting analysis was performed on SUM149-PT secondary MPS to separate ALDH1^high^ from ALDH1^low^ cells. 10,000 ALDH1^high^ and ALDH1^low^ cells were then cultured for 8 days under non-adherent conditions to form tertiary MPS. Cancer cells were labeled with 5 μM Cell Tracker Red CMTPX (Thermo Fisher Scientific, #C34552) for 1 h and the MPS area was measured using the *NIH Image-J* software. Graph showing the average of ALDH1^High^ and ALDH1^low^ MPS area from three independent experiments (+/− s.d.). **f** GFP-tagged SUM149-PT cells were cultured under non-adherent conditions for 24 days (three serial passages) to form tertiary mammospheres (MPS). In total, 10,000 SUM149-PT cells derived from tertiary MPS were then treated with DMSO (control) or 1 μM A37 (ALDH inhibitor) for 8 days. MPS area was measured using the *NIH Image-J* software. Graph showing the average of MPS area from three independent experiments (±s.d.).

To further explore the role of AURKA in inducing the enrichment of ALDH1^high^ TNBC cells, we employed the SUM149-PT cell line (basal-like phenotype) that was established from a metastatic inflammatory TNBC [4]. SUM149-PT cells showed TGFβR1 overexpression and higher levels of phosphorylated SMAD3 and AURKA compared to MDA-MB 231 cells (Fig. 2a). SUM149-PT cells also showed bone morphogenic protein 2 (BMP2) expression, while MDA-MB 231 cells expressed BMP1 (Supplementary Fig. 4a). Treatment of SUM149-PT cells with TGF-β1 did not significantly increase the percentage of ALDH1^high^ cells, indicating that TGFBR1 overexpression may promote a ligand-independent activation of the TGF-β signaling pathway (Fig. 2b, c). Nevertheless, genetic and pharmacologic AURKA targeting induced a significant decrease of ALDH1 activity (Fig. 2b, c), corroborating the essential role of AURKA expression and kinase activity in promoting the enrichment of ALDH1^high^ cells. Moreover, AURKA expression was also necessary to induce the expression of CD44 breast cancer stem-like cells marker (Supplementary Fig. 4b).

To define the mechanistic link between AURKA expression and self-renewal capacity that characterize BTICs, SUM149-PT cells were grown under non-adherent conditions to form tertiary mammospheres (MPS) that represent an in vitro surrogate assay of BTIC’s self-renewal capacity [36]. After 48 h, MPS were infected with scrambled lenti-shRNAs or lenti-shRNAs targeting AURKA mRNA and their growth was monitored for 8 days. AURKA genetic targeting impaired MPS growth, demonstrating the critical function of the AURKA signaling pathway in promoting cancer cells' self-renewal capacity (Fig. 2d). To investigate the extent to which high ALDH1 activity was necessary to maintain cancer cells' self-renewal capacity, FACS sorting analysis was performed on SUM-149PT secondary MPS to separate ALDH1^high^ from ALDH1^low^ cells. ALDH1^high^ and ALDH1^low^ cells were then cultured for 8 days under non-adherent conditions to form tertiary MPS. Tertiary MPS derived from ALDH1^low^ cells were significantly smaller than MPS derived from ALDH1^high^ cells (Fig. 2e). To determine the pivotal role of ALDH1 activity in mediating AURKA-induced self-renewal capacity, tertiary SUM-149PT MPS were treated with the potent ALDH1 inhibitor A37 for 8 days. A37 treatment significantly impaired SUM-149PT MPS growth (Fig. 2f). Taken together, these findings demonstrate the essential role of the AURKA/ALDH1 oncogenic axis in inducing cancer cells' self-renewal capacity that is required for MPS growth.

### AURKA targeting inhibits self-renewal capacity and restores sensitivity to DTX-based chemotherapy

**Fig. 3:**
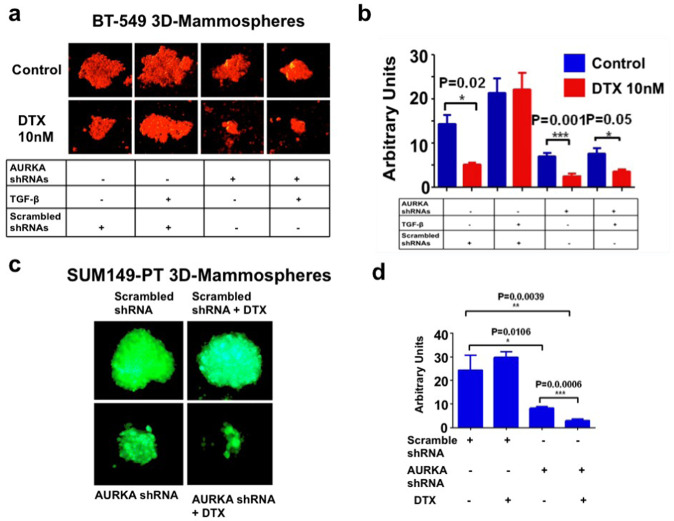
AURKA genetic targeting restores chemosensitivity. **a** BT-549 cells were cultured under non-adherent conditions for 24 days (three serial passages) to form tertiary mammospheres (MPS). In total, 10,000 cells derived from tertiary MPS were then infected with scrambled or AURKA lenti-shRNAs, treated with 10 ng/ml TGF-β1 in the presence of DMSO (control) or 10 nM docetaxel (DTX) and incubated for 8 days to monitor MPS growth. MPS were labeled with 5 μM Cell Tracker Red CMTPX (Thermo Fisher Scientific, #C34552) for 1 h and the MPS area was measured using the *NIH Image-J* software. **b** Graph showing the average of MPS area from three independent experiments (±s.d.). **c** GFP-tagged SUM149-PT cells were cultured under non-adherent conditions for 24 days (three serial passages) to form tertiary MPS. In total, 10,000 cells derived from tertiary MPS were then infected with scrambled or AURKA lenti-shRNAs and treated with DMSO (control) or 10 nM DTX for 8 days to monitor MPS growth. MPS area was measured using the *NIH Image-J* software. **d** Graph showing the average of MPS area from three independent experiments (±s.d.).

To define whether AURKA expression was necessary to mediate TGF-β-induced self-renewal capacity and chemoresistance, BT-549 tertiary MPS were infected with lenti-scrambled shRNAs or lenti-AURKA shRNAs and treated with TGF-β1 alone or in combination with 10 nM DTX for 8 days. While treatment of BT-549 MPS with lenti-scrambled shRNAs and TGF-β1 increased self-renewal capacity resulting in the emergence of chemoresistance, AURKA genetic targeting significantly inhibited TGF-β1-induced DTX resistance (Fig. 3a, b).

To further corroborate the pivotal role of AURKA expression in inducing chemoresistance, SUM149-PT TNBC cells were grown under non-adherent conditions to form tertiary MPS. After 48 h, SUM149-PT MPS were infected with lenti-scrambled shRNAs or lenti-AURKA shRNAs alone or in combination with 10 nM DTX and treated for 8 days. Inhibition of AURKA expression significantly impaired SUM-149PT MPS self-renewal capacity and restored DTX sensitivity (Fig. 2c, d). ALDH1 pharmacologic targeting also restored DTX sensitivity in SUM149-PT cells (Supplementary Fig. 4c), corroborating the pivotal role of ALDH1 activity in mediating AURKA-induced chemoresistance.

### AURKA expression is required to mediate TGF-β-induced SNAI1 regulatory network

**Fig. 4:**
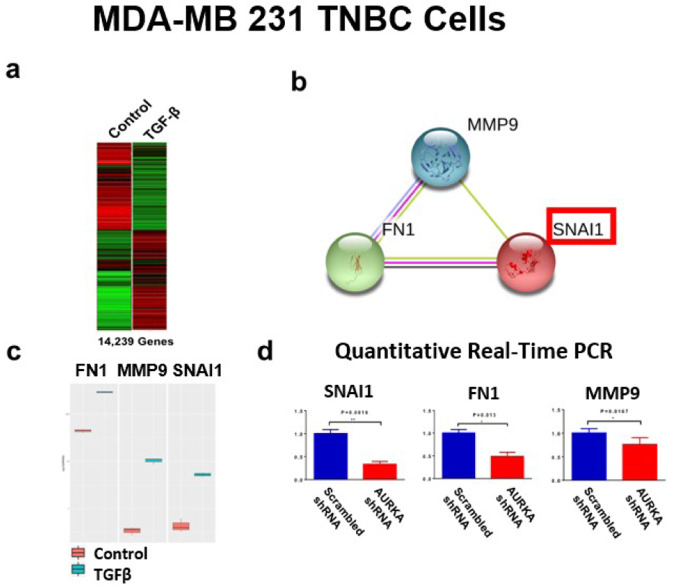
Transcriptomic analysis of MDA-MB 231 TNBC cells. **a** RNA-Seq analysis was performed on MDA-MB 231 cells treated with 10 ng/ml TGF-β1 for 48 h. 14,239 genes were differentially expressed between control and TGF-β1 groups. **b** *STRINGdb* software was used to identify a SNAI1/MMP9/FN1 Network. **c** Quantification analysis showing FN1, MMP9, and SNAI1 expression before and after TGF-β1 treatment. Experiments were performed in triplicate with a *p*-value < 0.05. **d** Real-time quantitative RT-PCR to detect SNA1, FN1, and MMP9 gene expression using MDA-MB 231 cells treated with 10 ng/ml TGF-β1 and infected with scrambled Lenti-shRNA or Lenti-shRNA targeting AURKA (*Origene*) for 48 h. Three independent experiments were performed in triplicate (±S.D. and *P-*value < 0.05).

To define the extent to which AURKA expression is required to mediate TGF-β-induced stemness reprogramming, MDA-MB 231 cells were treated with TGF-β1 and total RNA from untreated (control) and TGF-β1-treated samples were extracted to perform unbiased RNA-Seq analysis (Fig. 4a). Using *STRINGbd* software, we identified a SNAI1/FN1/MMP9 regulatory network that was up-regulated in MDA-MB 231 cells after treatment with TGF-β1 (Fig. 4b, c). Because SNAI1, FN1, and MMP9 play a key role in inducing EMT-associated cancer plasticity, chemoresistance, and metastasis, we aimed to define the pivotal role of AURKA in mediating TGF-β-induced expression of SNAI1, FN1, and MMP9 genes. MDA-MB 231 cells were infected with lenti-scrambled shRNAs or lenti-AURKA shRNAs and treated with TGF-β1 for 48 h. Quantitative real-time RT-PCR showed that AURKA genetic targeting significantly reduced the expression of SNAI1, FN1, and MMP9 genes (Fig. 4d).

### SNAI1 expression is necessary to induce ALDH1 activity, self-renewal capacity, and chemoresistance

**Fig. 5:**
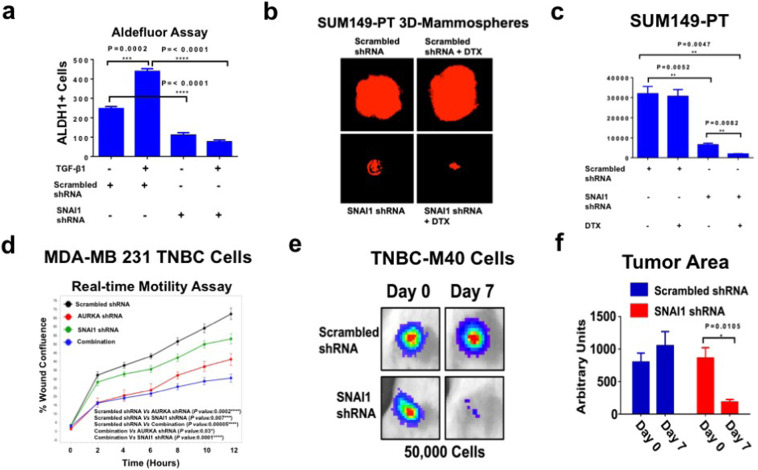
SNAI1 genetic targeting in TNBC cells. **a** BT-549 cells were treated with 10 ng/ml TGF-β1, scrambled lenti-shRNAs and lenti-shRNAs targeting SNAI1. After 48 h incubation, ALDH1 activity was detected with Aldefluor kit and measured by FACS analysis on 10,000 events. ALDH1 inhibitor DEAB was used as the control for each sample. Graph showing the average of ALDH1^High^ cells from three independent experiments (±s.d.). **b** SUM149-PT cells were cultured under non-adherent conditions for 24 days (three serial passages) to form tertiary MPS. In total, 10,000 cells derived from tertiary MPS were then infected with scrambled or SNAI1 lenti-shRNAs and treated with DMSO (control) or 10 nM DTX for 8 days to monitor MPS growth. MPS were labeled with 5 μM Cell Tracker Red CMTPX (Thermo Fisher Scientific, #C34552) for 1 h and MPS area was measured using the *NIH Image-J* software. **c** Graph showing the average of MPS area from three independent experiments (±s.d.). **d** In total, 12,000 cells infected with scrambled or AURKA and/or SNAI1 lenti-shRNAs were plated on 96-well costar plates and Real-time wound healing assay was performed by using the IncuCyte Instrument. Experiments were performed in triplicate (±s.d.). **e** In total, 50,000 TNBC-M40 cells infected with Luciferase lenti-vectors and scrambled Lenti-shRNA or SNAI1 scrambled Lenti-shRNA constructs were transplanted into the mammary fat pad of female NSG mice (three animals per group). Tumorigenic capacity was monitored in living animals at 0 (2 h post-injection was used as the control for cell viability) and 7 days post-injection by luciferase imaging. **f** The tumor Growth Area was quantified using *ImageJ-NIH* Software and represents the average of three animals per group.

Because the SNAI1 gene encodes for the SNAIL transcription factor that plays a central role in mediating TGF-β-induced EMT, stemness reprogramming, and tumor progression, BT-549 cells infected with scrambled lenti-shRNAs or lenti-SNAI1 shRNAs (Supplementary Fig. 4d) were treated with TGF-β1 and the enrichment of ALDH1^high^ cells was determined by aldefluor assay. Significantly, SNAI1 genetic targeting inhibited TGF-β-induced ALDH1 activity in BT-549 cells (Fig. 5a and Supplementary Fig. 5a), mimicking the effect of AURKA and SMAD3 targeting on impairing ALDH1 activity. To define the mechanistic link between SNAI1 expression, self-renewal capacity and chemoresistance, SUM149-PT cells, that expressed high levels of SNAIL protein (Supplementary Fig. 5b), were grown under non-adherent conditions to form tertiary MPS. After 48 h, SUM149-PT MPS were infected with lenti-scrambled shRNAs or lenti-SNAI1 shRNAs alone or in combination with 10 nM DTX and treated for 8 days. Inhibition of SNAI1 expression significantly impaired SUM-149PT MPS self-renewal capacity and restored DTX sensitivity (Fig. 5b, c). Since AURKA expression was required to induce a SNAI1/FN1/MMP9 invasive regulatory network, we aimed to establish the role of AURKA and SNAI1 in inducing motility of TNBC cells by performing an in vitro real-time wound healing assay. MDA-MB 231 cells were infected with scrambled lenti-shRNAs (control) or lenti-shRNAs targeting AURKA and/or SNAI1 mRNA and wound healing repair was monitored for 12 h. Genetic targeting of the AURKA/SNAI1 oncogenic axis significantly inhibited the motility capacity of MDA-MB 231 cells (Fig. 5d).

Finally, to define in vivo the extent to which reduced SNAI1 expression impaired tumorigenic capacity, we established unique TNBC xenografts using TNBC-M40 cells (Supplementary Fig. 5c) that were isolated from patient-derived TNBC brain metastasis. TNBC-M40 cells were infected with luciferase lenti-vectors and scrambled lenti-shRNAs (control) or SNAI1 lenti-shRNAs constructs. After 48 h infection, TNBC-M40 cells (50,000) were injected into the mammary fat pad of female NSG mice and tumorigenic capacity was assessed after 1-week injection using the Xenogen imaging system. SNAI1 genetic targeting significantly inhibited the tumorigenic capacity of TNBC-M40 cells (Fig. 5e, f). Taken together, these results demonstrate that SNAI1 expression is required to induce ALDH1 activity that is linked to self-renewal capacity, chemoresistance, and high tumorigenicity in TNBC cells.

### Dual TGF-β and AURKA pharmacologic targeting reduces SNAIL protein expression and induces apoptosis in TNBC cells

**Fig. 6:**
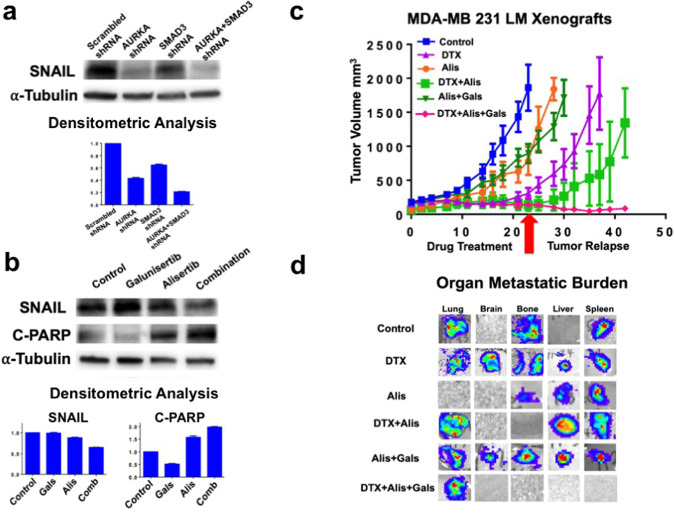
Dual pharmacologic targeting of TGF-β and AURKA oncogenic pathways. **a** Immunoblot assay showing SNAIL protein expression in MDA-MB 231 cells infected with scrambled lenti-shRNAs or lenti-shRNAs targeting SNAI1 for 48 h. Densitometry analysis showing the fold change of SNAIL protein levels in MDA-MB 231 TNBC cells normalized to α-Tubulin. Experiments were performed in triplicate. **b** Immunoblot assay showing SNAIL and cleaved-PARP expression in MDA-MB 231 cells treated with galunisertib (50 nM) and/or alisertib (50 nM) for 48 h. Densitometry analysis showing the fold change of SNAIL and cleaved-PARP protein levels in MDA-MB 231 TNBC cells normalized to α-Tubulin. Experiments were performed in triplicate. **c** Establishment of MDA-MB 231 LM xenografts: 1 × 10^6^ cells were injected into the mammary fat pad of 4 weeks old female NSG mice. After 2 weeks of tumor growth, mice were randomized into six groups (five animals each group) and treated with 10 mg/Kg DTX (IP injections), 50 mg/Kg galunisertib (oral gavage), and 50 mg/Kg alisertib (oral gavage) 3 times/week for 3 weeks. After drug treatment, tumor relapse was monitored for additional 3 weeks or when the tumor xenografts reached a volume comparable to control groups. Tumor volume was measured 3 times/week using a digitalized caliper. **d** Following drug treatment and tumor relapse, mice were sacrificed and organ metastatic burden was determined ex-vivo using the Xenogen imaging system.

To define whether AURKA and SMAD3 genetic targeting reduced SNAIL protein expression, MDA-MB 231 cells were infected with scrambled lenti-shRNAs (control) or lenti-shRNAs targeting AURKA and/or SMAD3 mRNA. AURKA targeting was more effective in reducing SNAIL expression than SMAD3 targeting. However, the combination of AURKA and SMAD3 lenti-shRNAs resulted in the strongest reduction of SNAIL expression (Fig. 6a). To define whether dual pharmacologic targeting of TGF-β and AURKA pathways could result in a better therapeutic efficacy due to SNAIL down-regulation, MDA-MB 231 cells were treated with galunisertib (TGF-β inhibitor) and/or alisertib (AURKA inhibitor). Significantly, the combination of galuniserib and alisertib induced the highest reduction of SNAIL expression that was linked to the highest levels of cleaved PARP used as a marker of apoptosis (Fig. 6b).

### Combination of DTX with dual TGF-β/AURKA-targeted therapy inhibits tumor relapse and the emergence of distant metastasis

To establish in vivo the therapeutic efficacy of dual TGF-β/AURKA-targeted therapy in combination with DTX, we used highly metastatic MDA-MB 231 LM cells infected with lenti-vectors expressing luciferase to monitor tumor growth in living animals as previously described [46]. MDA-MB 231 LM cells were injected into the mammary fat pad of female NSG mice and two weeks after tumor growth, animals were randomized into six groups and treated with DTX, alisertib, and galunisertib for three weeks (Fig. 6c). Following drug treatments, tumor relapse was monitored for additional 3 weeks to detect the emergence of distant metastasis. Animals were euthanized and organs (lung, brain, bone, liver, spleen) were removed and assayed for the presence of cancer cells using the Xenogen imaging system. Significantly, the combination of DTX, galunisertib, and alisertib was effective in impairing tumor relapse that was linked to inhibition of metastatic burden (Fig. 6d and Table 1).

**Table 1:** Organ metastatic burden: following drug treatment and tumor relapse, mice were sacrificed and organ metastatic burden was determined ex-vivo using the Xenogen imaging system by counting the number of organs that showed metastasis.

Drug treatment	Lung	Brain	Bone	Liver	Spleen	Organ metastatic burden	
CTR	4/5	0/5	5/5	0/5	1/5	10/25	40%
DTX	2/5	1/5	2/5	1/5	2/5	8/25	32%
Alis	0/5	0/5	1/5	2/5	2/5	5/25	20%
Alis + DTX	3/5	0/5	0/5	3/5	4/5	10/25	40%
Alis + Gals	5/5	1/5	4/5	1/5	2/5	13/25	52%
Alis + Gals + DTX	3/5	0/5	0/5	0/5	0/5	3/25	12%

### Dual TGF-β and AURKA pharmacologic targeting inhibits ALDH1 activity, self-renewal capacity and enhances chemosensitivity in TNBC cells isolated from metastatic PDXs

**Fig. 7:**
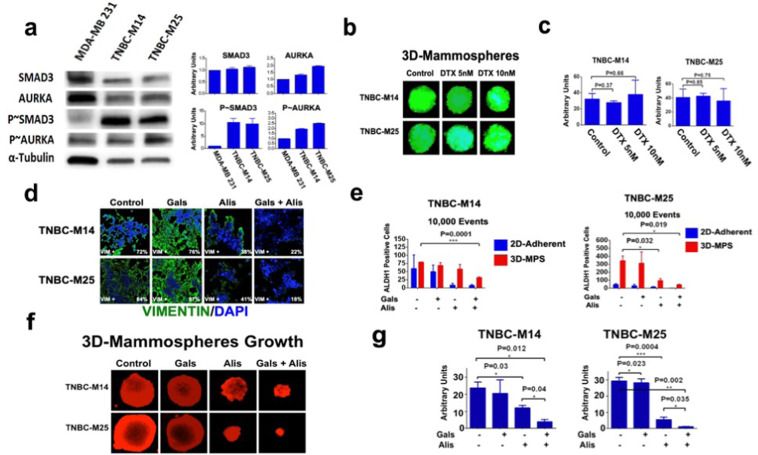
Dual targeting of TGF-β and AURKA oncogenic pathways inhibits TNBC plasticity. **a** Immunoblot analysis showing higher phospho-SMAD3 and phospho-AURKA in TNBC-M14 and TNBC-M25 tertiary MPS compared to MDA-MB 231 used as control. Graphs showing the densitometric quantification of total and phosphoproteins expression normalized to Tubulin. **b** GFP-tagged TNBC-M14 and TNBC-M25 cells were cultured under non-adherent conditions for 24 days (three serial passages) to form tertiary MPS. In total, 10,000 cells derived from tertiary MPS were then treated with DMSO (control) or DTX for 8 days to monitor MPS growth. MPS area was measured using the *NIH Image-J* software. **c** Graph showing the average of MPS area from three independent experiments (±s.d.). **d** TNBC-M14 and TNBC-M25 cells were treated with 50 nM galunisertib, 50 nM alisertib, and combination for 48 h. Expression and cellular localization of Vimentin (green) was assessed by Immunofluorescence employing the *Zeiss Confocal Fluorescent Microscope*. Experiments were performed in triplicate with similar results. **e** Adherent and mammospheres-derived TNBC-M14 and TNBC-M25 were treated with 50 nM galunisertib (Gala), 50 nM alisertib (Alis), and combination. After 48 h incubation, ALDH activity was measured by FACS analysis on 10,000 events. ALDH inhibitor DEAB was used as control. Experiments were performed in triplicate (±s.d.). **f** TNBC-M14 and TNBC-M25 cells were cultured under non-adherent conditions for 24 days (three serial passages) to form tertiary MPS. In total, 10,000 cells derived from tertiary MPS were then treated with 50 nM galunisertib, 50 nM alisertib, and combination for 8 days. MPS were labeled in red using the *CellTracker Red CMTPX Dye*. **g** MPS area was measured using the *NIH Image-J* software. Experiments were performed in triplicate (±S.D. and *P*-value < 0.005).

To overcome the limited translatability of established breast cancer cell lines and corroborate the role of the TGF-β/AURKA oncogenic axis in driving cancer plasticity and chemoresistance in clinically relevant models, we established unique TNBC-M14 and TNBC-M25 (Supplementary Fig. 5c) isolated from patient-derived TNBC brain metastasis. TNBC-M14 and TNBC-M25 cells showed higher levels of phosphorylated SMAD3 and AURKA compared to MDA-MB 231 cells (Fig. 7a), making them suitable models for dual pharmacologic targeting of TGF-β and AURKA oncogenic pathways. To define whether TNBC-M14 and TNBC-M25 models showed high self-renewal capacity and intrinsic chemoresistance, we developed GFP-tagged TNBC-M14 and TNBC-M25 tertiary MPS. After 48 h incubation, TNBC-M14 and TNBC-M25 MPS were treated with either 5 nM or 10 nM DTX for 8 days. TNBC-M14 and TNBC-M25 MPS growth was not impaired by DTX treatment, indicating their intrinsic resistance to DTX-based chemotherapy (Fig. 7b, c).

Next, we investigated the extent to which TNBC-M14 and TNBC-M25 chemoresistant phenotype were linked to TGF-β/AURKA axis-induced cancer plasticity. TNBC-M14 and TNBC-M25 cells were treated with galunisertib, alisertib, and combination for 48 h. Combinatorial treatment with galunisertib and alisertib showed the lowest number of TNBC cells expressing the mesenchymal marker vimentin (Fig. 7d). However, galunisertib and alisertib did not restore the expression of the epithelial marker E-cadherin (data not shown). To establish whether dual TGF-β and AURKA pharmacologic targeting were also effective in reducing the enrichment of ALDH1^high^ cells, we performed an Aldefluor assay in TNBC-M14 and TNBC-M25 tertiary MPS treated with galunisertib, alisertib and combination (2D-cultured cells were used as control). Although alisertib monotherapy reduced ALDH1 activity in TNBC-M25 MPS, the combination of galunisertib and alisertib was effective in reducing ALDH1 activity in both TNBC-M14 and TNBC-M25 MPS (Fig. 7e and Supplementary Fig. 6). ALDH1 pharmacologic targeting also enhanced the therapeutic efficacy of alisertib in impairing cell proliferation (Supplementary Fig. 7a).

To assess the effect of the combinatorial treatment in impairing self-renewal capacity and MPS growth, TNBC-M14 and TNBC-M25 cells were grown under non-adherent conditions to form tertiary MPS. After 48 h, TNBC-M14 and TNBC-M25 MPS were treated with galunisertib alone, alisertib alone, and the combination for 8 days. While TNBC-M14 and TNBC-M25 MPS were resistant to galunisertib monotherapy, alisertib monotherapy was sufficient to inhibit MPS growth in both TNBC models. Nevertheless, the combination of galunisertib and alisertib resulted in the strongest inhibition of TNBC-M14 and TNBC-M25 MPS self-renewal capacity (Fig. 7f, g).

**Fig. 8:**
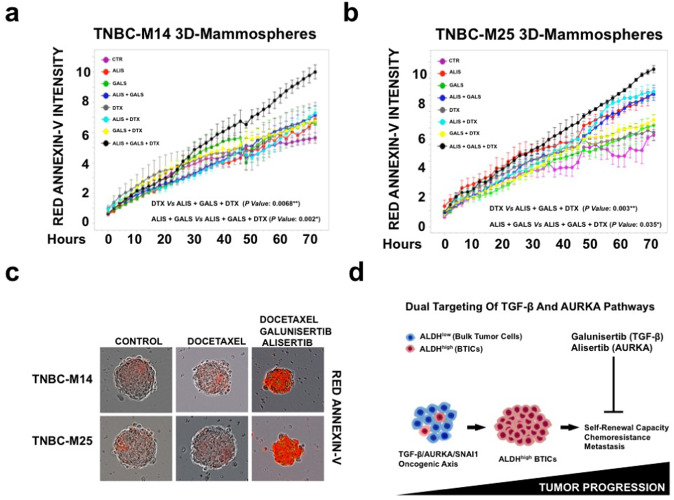
Dual targeting of TGF-β and AURKA pathways enhances the sensitivity to docetaxel-based chemotherapy. **a**, **b** Real-time apoptosis assay of TNBC-M14 and TNBC-M25 3D-MPS treated with 10 nM Docetaxel, 50 nM Galunisertib, and 50 nM Alisertib as single agents and in combination. Apoptotic cells were stained in red with ANNEXIN-V and quantified using the *Cell Player System* (IncuCyte, BioEssen). Experiments were performed in triplicate (±S.D. and *P-*value < 0.05). **c** Representative images of TNBC-M14 and TNBC-M25 3D-MPS treated with 10 nM Docetaxel, 50 nM Galunisertib, and 50 nM Alisertib as single agents and in combination. Apoptotic cells were stained in Red with ANNEXIN-V and quantified in real-time using the *Cell Player System* (IncuCyte, BioEssen). **d** TGF-β/AURKA oncogenic axis promotes the enrichment of chemoresistant ALDH^high^ BTICs: Bulk TNBC cells show a chemosensitive ALDH^low^ phenotype. Aberrant activation of TGF-β/AURKA/SNAI1 oncogenic axis induces TNBC plasticity resulting in the enrichment of ALDH1^high^ BTICs with intrinsic resistance to standard chemotherapeutic agents. Dual pharmacologic inhibition of TGF-β and AURKA pathways will impair TNBC plasticity and restore chemosensitivity through the selective targeting of ALDH1^high^ BTICs.

To evaluate the extent to which dual TGF-β and AURKA pharmacologic targeting were effective in reversing DTX resistance, we established TNBC-M14 and TNBC-M25 tertiary MPS and performed a real-time assay using Annexin-V as a marker of apoptosis. TNBC-M14 and TNBC-M25 MPS were treated with DTX, alisertib, and galunisertib monotherapy and combinations. While TNBC-M14 and TNBC-M25 MPS showed nominal sensitivity to DTX monotherapy, the combination of DTX, galunisertib, and alisertib resulted in the strongest induction of apoptosis (Fig. 8a–c and Supplementary Fig. 7b). Taken together, these findings demonstrate that dual pharmacological targeting of TGF-β and AURKA signaling pathways was effective in impairing the self-renewal capacity of chemoresistant TNBC cells that were linked to an enhancement of the therapeutic response to DTX-based chemotherapy.

## Discussion

Breast cancer represents the second largest cause of cancer-related mortality among women worldwide [47]. TNBCs represent the most aggressive subtype of this malignancy and the mainstay of treatment remains chemotherapy because of few FDA-approved targeted therapies [45]. Cytotoxic chemotherapeutic agents such as taxanes are initially effective in TNBC patients and significantly reduce tumor burden [48]. Unfortunately, the majority of TNBCs relapse after standard chemotherapy, and metastatic lesions exhibit drug resistance that limits the effectiveness of therapy and the survival of patients with advanced TNBC [49]. Several studies strongly indicate that early tumor relapse and progression is functionally linked to the enrichment of a sub-fraction of cancer cells termed BTICs that undergo EMT-mediated cancer plasticity and typically exhibit a basal-like CD44^+^/CD24^-^ and/or ALDH1^high^ phenotype with critical cancer stem-like features such as high self-renewal capacity and intrinsic resistance to chemotherapy [50]. One of the major mechanisms responsible for the intrinsic drug resistance of BTICs is their high ALDH1 activity leading to inhibition of chemotherapy-induced apoptosis [51]. TGF-β signaling is a well-characterized oncogenic pathway that promotes EMT-mediated cancer plasticity and tumor stemness in TNBC. Activation of TGF-β signaling induces the expression of EMT-ATFs that function as molecular switches for the EMT and stemness nuclear reprogramming. SNAI1 gene encodes for SNAIL protein, a major EMT-ATF transcriptional factor that plays a key role in inducing, cancer cell plasticity, chemoresistance and metastasis [52]. It has been demonstrated in experimental TNBC models that treatment with the chemotherapeutic agent paclitaxel induced the expansion of drug-resistant BTICs and restoration of chemosensitivity was achieved only after pharmacologic inhibition of TGF-β signaling [53]. However, long-term administration of TGF-inhibitors has been associated with the outgrowth of chemoresistant breast cancer cells [54]. These findings suggest that following pharmacologic inhibition of TGF-β signaling, highly plastic tumors can activate alternative escape oncogenic pathways that will by-pass the action of TGF-β-targeted monotherapy, propagating a chemoresistant phenotype. Due to the current limitations of TGF-β-targeted monotherapy, it is expected that TGF-β inhibitors will show their best therapeutic activity in combination with other targeted agents to reverse chemoresistance and delay tumor progression. Therefore, the molecular characterization of key oncogenic pathways that mediate TGF-β-induced cancer cell plasticity and chemoresistance is mandatory to develop effective combinatorial targeted therapies for advanced TNBC.

In this study, we aimed to define whether TGF-β-induced cancer cell plasticity and enrichment of chemoresistant ALDH1^high^ TNBC cells were mediated by the mitotic kinase AURKA that also plays a key role in tumor progression [36, 37]. Treatment of TNBC cells with TGF-β1 induced AURKA phosphorylation that was linked to enrichment of ALDH1^high^ TNBC cells. AURKA targeting impaired the enrichment of ALDH1^high^ TNBC cells and their self-renewal capacity regardless of TGF-β1 stimulation. SMAD3 targeting also impaired TGF-β-induced ALDH1 activity, demonstrating that TGF-β induces the enrichment of ALDH1^high^ TNBC cells through the canonical SMAD3 and non-canonical AURKA signaling pathways. Since AURKA promotes a chemoresistant cancer phenotype [37], we investigated whether TGF-β-induced chemoresistance was also dependent on AURKA expression. AURKA genetic targeting reversed resistance to DTX and stimulation with TGF-β1 did not rescue a chemoresistant phenotype. Taken together, these results demonstrated that TGF-β-induced ALDH1 activity, self-renewal capacity and DTX resistance are strictly dependent on AURKA expression in TNBC. Furthermore, these findings are in agreement with our previous studies that showed the central role of AURKA in conferring to breast cancer cells a chemoresistant phenotype [37]. Mechanistically, AURKA mediates TGF-β-induced ALDH1 activity, self-renewal capacity and chemoresistance by promoting SNAIL expression. AURKA and SMAD3 genetic targeting reduced SNAIL expression, providing the therapeutic rationale to co-target TGF-β and AURKA signaling pathways in TNBC. Because TGF-β and AURKA signaling pathways provide attractive druggable targets to halt tumor progression, we developed tumor xenografts to define in vivo the efficacy of dual TGF-β/AURKA targeted therapy in combination with DTX. Remarkable, a combination of galunisertib (TGF-β inhibitor), alisertib (AURKA inhibitor), and DTX inhibited tumor relapse and organ metastatic burden. These results indicate that combination of dual TGF-β/AURKA targeted therapy with standard chemotherapy is required for efficient eradication of bulk tumor cells. We also employed unique TNBC cells (TNBC-M14 and TNBC-M25) isolated from patient-derived TNBC brain metastasis that exhibited high endogenous activation of TGF-β and AURKA pathways. TNBC-M14 and TNBC-M25 MPS showed intrinsic resistance to DTX that was linked to high ALDH1 activity and self-renewal capacity. TNBC-M14 and TNBC-M25 cells expressed the mesenchymal marker vimentin and combinatorial treatment with galunisertib and alisertib strongly reduced vimentin expression, demonstrating that TGF-β and AURKA signaling pathways are required to promote an invasive mesenchymal-like phenotype. Significantly, dual pharmacologic targeting of TGF-β and AURKA pathways also reduced the enrichment of ALDH1^high^ TNBC cells that was linked to inhibition of MPS growth and restoration of DTX sensitivity. Nevertheless, treatment with alisertib monotherapy was more effective than galunisertib monotherapy, corroborating our findings that AURKA mediates TGF-β-induced TNBC progression and aberrant AURKA activation may represent a major mechanism of resistance to small molecule inhibitors of TGF-β signaling pathway.

In this study, highlights a novel cross-talk between TGF-β and AURKA oncogenic pathways responsible for the emergence of chemoresistance and TNBC progression: ALDH1^low^ bulk tumor cells with nominal AURKA expression/activity show low self-renewal capacity and sensitivity to the standard of care chemotherapy. Aberrant AURKA expression/activity is required to promote tumor progression because of its central role in mediating TGF-β-induced SNAI1 gene expression, cancer cell plasticity, and the enrichment of ALDH1^high^ cells harboring high self-renewal capacity and intrinsic chemoresistance. Conversely, dual pharmacologic targeting of TGF-β and AURKA pathways efficiently reverses chemoresistance and impairs tumor progression through SNAI1 down-regulation, inhibition of cancer cell plasticity, and selective targeting of ALDH1^high^ cells (Fig. 8d). These findings provide the strong preclinical rationale to launch novel combinatorial therapeutic strategies tailored for the clinical management of metastatic TNBCs that are refractory to chemotherapy.

## Materials and methods

### METABRIC analysis

Breast tumor specimens were selected from participants of the Molecular Taxonomy of Breast Cancer International Consortium (METABRIC) public database (details are provided in Supplementary information).

### Established breast cancer cell lines

Details are provided in Supplementary information.

### Patient-derived TNBC cells

Details are provided in Supplementary information.

### Immunoblot, immunofluorescence, and FACS sorting assays

Immunoblot, Immunofluorescence, and FACS sorting assays were performed as previously described [36, 37]. Details are provided in Supplementary information.

### Lenti-vectors targeting AURKA, SMAD3, and SNAI1

Scrambled (control), AURKA (Origene, TL320538), SMAD3 (Origene, TL309254), and SNAI1 shRNA lenti-vectors (Origene, TL309226) were purchased by OriGene Technologies (Rockville, MD, USA) and used according to manufacturer’s instructions.

### Mammospheres formation, chemoresistance, real-time apoptosis, and proliferation assays

Details are provided in Supplementary information.

### ALDH1 activity assay

Details are provided in Supplementary information.

### Total RNA isolation and RNA-seq studies

Total RNA was isolated from MDA-MB 231 TNBC cells using a total RNA Ready-To-Use kit (BioChain). Details are provided in Supplementary information.

### Tumor xenografts

Details are provided in Supplementary information.

### Scientific rigor and statistical analysis

Details are provided in Supplementary information.

## Supplementary information

Related Manuscript File

SFIGURE 1

SFIGURE 2

SFIGURE 3

SFIGURE 4

SFIGURE 5

SFIGURE 6

SFIGURE 7
