# Supplementary material for: Aurora-A kinase oncogenic signaling mediates TGF-β-induced triple-negative breast cancer plasticity and chemoresistance
Source: Oncogene. 2021 Mar 5;40(14):2509–23. doi: 10.1038/s41388-021-01711-x (PMC8032554; doi:10.1038/s41388-021-01711-x)
Supplement: Supplementary file 1 — Related Manuscript File [file 41388_2021_1711_MOESM1_ESM.docx]

**Jalalirad at al.**

**Supplementary Information**

**Aurora-A Kinase Oncogenic Signaling Mediates TGF-β-Induced Triple Negative Breast Cancer Plasticity and Chemoresistance**

Mohammad Jalalirad, Tufia C. Haddad, Jeffrey L. Salisbury, Derek Radisky, Minzhi Zhang, Mark Schroeder, Ann Tuma, Eduard Leof, Jodi M. Carter, Amy C. Degnim, Judy C. Boughey, Jann Sarkaria, Jia Yu, Liewei Wang, Minetta Liu, Luca Zammataro, Lorenzo Malatino, Evanthia Galanis, James N. Ingle, Matthew P. Goetz and Antonino B. D’Assoro

**Supplementary Material and Methods**

**METABRIC Analysis:** The METABRIC database ([http://molonc.bccrc.ca/aparicio lab/research/metabric/](http://molonc.bccrc.ca/aparicio-lab/research/metabric/)) contains clinical traits, expression, copy number variation profiles and single nucleotide polymorphism (SNP) genotypes derived from breast cancer patients.

**Established Breast Cancer Cell Lines:** The human breast cancer cell lines BT-549 and MDA-MB 231 were obtained from ATCC (Manassas, VA, USA). SUM149-PT cancer cells were kindly provided by Dr. Couch’s laboratory (Mayo Clinic, Rochester, MN, USA). All cell lines were maintained in DMEM medium containing 5mM glutamine, 1% penicillin/streptomycin and 10% FBS at 37 C in 5% CO2 atmosphere. All cell lines were tested for mycoplasma contamination.

**Patient-Derived TNBC Cells:** TNBC-M14, TNBC-M25 and TNBC-M40 cells were isolated from patient-derived brain metastasis TNBC xenograft models that were generated at the Mayo Clinic Cancer Center [45]. To establish cultured TNBC-M14, TNBC-M25 and TNBC-M40 cells, patient-derived xenograft metastatic models were excised from killed animals, minced using sterile scissors, transferred to complete culture medium and fibroblast-free TNBC-M14, TNBC-M25 and TNBC-M40 cells were cultured in low-adherent flasks supplemented with MammoCult^TM^ medium (STEMCELL 05620) and propagated in culture as 3D-Mammospheres and used for this study.

**Immunoblot, Immunofluorescence and FACS Sorting Assays:** Antibodies employed to perform these studies were the followings: AURKA (AbCam 13824, Cambridge, MA, USA); phospho-Thr288 AURKA (Cell Signalling 3079, Beverly, MA, USA); TGF beta Receptor I (AbCam 31013, Cambridge, MA, USA ); TGF beta Receptor II (AbCam 184948, Cambridge, MA, USA); SMAD3 (AbCam 40854, Cambridge, MA, USA); phospho-S423/S425 SMAD3 (AbCam 52903, Cambridge, MA, USA); E-Cadherin (Santa Cruz Biotechnology, sc-8426); vimentin (AbCam 52903, Cambridge, MA, USA); α-tubulin (Sigma T9026, [St. Louis, Missouri](http://en.wikipedia.org/wiki/St._Louis,_Missouri), USA, ); SNAIL (AbCam 216347, Cambridge, MA, USA). FITC and Rodhamine secondary antibodies were obtained from Molecular Probes (Eugene, OR, USA).

**Mammospheres Formation:** 10,000 TNBC cells derived from secondary mammospheres were plated in ultra-low attachment 96-well culture dishes in 100 µL of MammoCult^TM^ medium (STEMCELL 05620) to form tertiary mammospheres and medium was added every 48 hours for a maximum of 8 days. Mammospheres growth was recorded through a digital camera (Nikon).

**Chemoresistance Assay:** 5mg Docetaxel (DTX), 5mg Galunisertib (GALS) and 5mg Alisertib (ALIS) were purchased from Selleckem and diluted in DMSO as 10mM stock solution. 10mg A37 was purchased from Tocris and diluted in DMSO as 10mM stock solution. Breast cancer cells were plated in 2D-cultures supplemented with DMEM medium or ultra-low attachment 96-well culture dishes in 100 µL of MammoCult^TM^ medium (STEMCELL 05620) to form tertiary mammospheres and medium with DTX, galunisertib, alisertib and A37 was replaced every 48 hours.

**Real-Time Apoptosis Assay:** 10,000 TNBC-M14 and TNBC-M25 cells derived from secondary mammospheres were plated in ultra-low attachment 96-well culture dishes in 100 µL of MammoCult^TM^ medium (STEMCELL 05620) to form tertiary mammospheres. After 24 hours, cells were treated with DTX, galunisertib and alisertib (alone or in combination) and incubated for additional 72 hours in the presence of Red ANNEXIN-V (Essen BioScience 4641). Apoptotic cells were quantified in real-time using *IncuCyte S3* (Essen BioScience).

**Real-Time Cell Proliferation Assay:** 10,000 SUM149-PT TNBC cells were plated in 96-well culture dishes in 100 µL of DMEM medium to form tertiary mammospheres. After 24 hours, cells were treated with A37, galunisertib and alisertib (alone or in combination) and incubated for 6 days. Cell proliferation was quantified in real-time using *IncuCyte S3* (Essen BioScience).

**ALDH1 Activity Assay:** ALDH1 activity was detected by FACS analysis using the Aldefluor assay kits (STEMCELL Technologies 01700, Canada; Millipore Sigma SCR150, USA) according to the manufacturer's instructions.

**Total RNA Isolation and RNA-Seq Studies:** Library preparation for RNA-Seq and Bioinformatics analysis was performed at the Genomic Analysis Core (Mayo Clinic, Rochester, MN). RNA-Seq data are available at GSE160990: [http://www.ncbi.nlm.nih.gov/geo/query/acc.cgi?acc=GSE160990](http://www.ncbi.nlm.nih.gov/geo/query/acc.cgi?acc=GSE160990)**.**

**Tumor Xenografts:** Procedures established by the Institutional Animal Care and Use Committee based on US NIH guidelines for the care and use of laboratory animals were followed for all experiments. Establishment of MDA-MB 231 LM xenografts: 4 weeks old non-ovariectomized female NSG mice (the Jackson Laboratory) were anesthetized by exposure to 3% isoflurane and injected into the mammary fat pad with 1× 106 cells (infected with a luciferase lenti-vector to detect the presence of distant metastasis) suspended in 50 ul of 50% Matrigel (BD Bioscience, Bedford, MA, USA). After 2 weeks tumor growth, mice were randomized into six groups (5 animals each group) and treated with 10mg/Kg DTX (IP injections), 50mg/Kg galunisertib (oral gavage) and 50mg/Kg alisertib (oral gavage) 3 times/week for 3 weeks. After drug treatment, tumor relapse was monitored for additional 3 weeks or when the tumor xenografts reached a volume comparable to control groups. Tumor volume was measured 3 times/week using a digitized caliper. Following drug treatment and tumor relapse, mice were sacrificed and organ metastatic burden was determined *ex-vivo* using the Xenogen imaging system.

**Scientific Rigor and Statistical Analysis:** Quantitative Real-Time RT-PCR, FACS, immunoblot and Immunofluorescence assays were run in triplicate or for 3 independent runs (*+/- S.D.*). The average read-out of the triplicates from each run was determined and a 95% t-confidence interval for the difference between was constructed. Different triple-negative breast cancer cell lines and patient-derived xenografts (PDXs) were employed in this study to increase the power to detect the effect size. The nonparametric Mann-Whitney t test (Statview software) was used to determine the significance of the relative tumor volumes for treated versus untreated TNBC xenograft groups. Using an initial sample size of 5 animals per group and eventually increases this to a sample size of 10 animals per group only if experimental outcomes warrant it, a two-sided (alpha=0.05), two sample t-test for assessing whether the difference in mean tumor burden differs significantly between a particular pair of treatment groups will have a power of 90% to detect a difference of 1.6 standard deviation (*SD*). For each xenograft (treated and control groups), the difference in the percentage of organ metastatic burden was assessed*.* Animals were examined every day and body weight and primary tumor size was measured 3 times per week.

**Supplementary Figure Legend**

**Supplementary Figure 1:** METABRIC survival analysis of ER+, PR+, HER-2- and ER-, PR-, HER-2+ subgroups showing that aberrant AURKA expression was not significantly associated with reduced patient overall survival.

**Supplementary Figure 2: (a)** BT-549 cells were treated with 10ng/ml TGF-β1 and lenti-shRNAs. After 48 hours incubation, ALDH1 activity was detected with Aldefluor kit and measured by FACS analysis on 10,000 events. ALDH1 inhibitor DEAB was used as control for each sample.  **(b)** MDA-MB 231 cells were treated with 10ng/ml TGF-β1 and lenti-shRNAs. After 48 hours incubation, ALDH1 activity was detected with Aldefluor kit and measured by FACS analysis on 10,000 events. ALDH1 inhibitor DEAB was used as control for each sample. **(c)** Graph showing the average of ALDH1^high^ cells from three independent experiments (+/- s.d.).

**Supplementary Figure 3: (a)** Immunofluorescence analysis showing BT-549 cells treated with 10ng/ml TGF-β1 and lenti-shRNAs. After 72 hours incubation, cells were stained for vimentin (green) and E-cadherin (red) EMT markers. Nuclei were labeled in blue with DAPI. **(b)** Immunofluorescence analysis showing BT-549 cells treated with 10ng/ml TGF-β1. After 48 hours incubation, cells were stained for centrin (green) and pericentrin (red) centrosome markers. **(c)** Immunoblot analysis showing SMAD3 expression in BT-549 cells infected with scrambled and SMAD3 shRNA lenti-vectors. SMAD3 shRNA (B) showed the highest reduction of SMAD3 expression and was used in this study. Graphs showing the densitometric quantification of SMAD3 normalized to Lamin B1 . **(d)** BT-549 cells were treated with 10ng/ml TGF-β1 and lenti-shRNAs. After 48 hours incubation, ALDH1 activity was detected with Aldefluor kit and measured by FACS analysis on 10,000 events. ALDH1 inhibitor DEAB was used as control for each sample.  **(e)** Immunoblot analysis showing nuclear phosphorylated SMAD3 expression in BT-549 cells treated with 10ng/ml TGF-β1 and/or alisertib. Graphs showing the densitometric quantification of SMAD3 expression normalized to Laminin.

**Supplementary Figure 4: (a)** Immunoblot analysis showing BMP1 and BMP2 expression in MDA-MB 231 and SUM149-PT cells. Tubulin expression was used as protein loading control **(b)** Immunofluorescence analysis showing CD44 expression (red) in SUM149-PT cells infected with scrambled and AURKA shRNA lenti-vectors. Nuclei were labeled in blue with DAPI. **(c)** Real-time apoptosis assay of SUM149-PT cells treated with 10 nM DTX and/or 1 uM A37. Apoptotic cells were stained in Red with ANNEXIN-V and quantified using the *Cell Player System* (IncuCyte, BioEssen). Experiments were performed in triplicate *(+/- S.D. and P value < 0.05*). **(d)** Immunoblot analysis showing SNAIL expression in BT-549 cells infected with scrambled and SNAI1 shRNA lenti-vectors. SNAI1 shRNA (B) showed the highest reduction of SNAIL expression and was used in this study. Graphs showing the densitometric quantification of SNAIL expression normalized to Tubulin.

**Supplementary Figure 5: (a)** BT-549 cells were treated with 10ng/ml TGF-β1 and lenti-shRNAs. After 48 hours incubation, ALDH1 activity was detected with Aldefluor kit and measured by FACS analysis on 10,000 events. ALDH1 inhibitor DEAB was used as control for each sample. **(b)** Immunoblot analysis showing SNAIL expression in MDA-MB 231and SUM149-PT cells. Graph showing the densitometric quantification of SNAIL expression normalized to Tubulin. **(c)** Immunofluorescence analysis showing expression of ER (red), PR (green) and HER-2 (red) in BT-474, TNBC-M14, TNBC-M25 and TNBC-M40 cells. Nuclei were stained in blue with DAPI.

**Supplementary Figure 6:** TNBC-M14 and TNBC-M25 3D-MPS treated with 50nM Galunisertib and 50nM Alisertib as single agents and in combination. After 48 hours incubation, ALDH1 activity was detected with Aldefluor kit and measured by FACS analysis on 10,000 events. ALDH1 inhibitor DEAB was used as control for each sample. Graph showing the average of ALDH1^high^ cells from three independent experiments (+/- s.d.).

**Supplementary Figure 7: (a)** Cell proliferation assay of SUM149-PT cells treated with 1 uM A37 as monotherapy or in combination with 50 nM galunisertib or 50 nM alisertib. Cell proliferation was monitored in real-time and quantified using the *Cell Player System* (IncuCyte, BioEssen). Experiments were performed in triplicate. **(b)** Representative images of TNBC-M14 and TNBC-M25 3D-MPS treated with 10 nM Docetaxel, 50nM Galunisertib and 50nM Alisertib as single agents and in combination. Apoptotic cells were stained in Red with ANNEXIN-V and quantified in real-time using the *Cell Player System* (IncuCyte, BioEssen).
