# Supplementary figures and images for: Aurora-A kinase oncogenic signaling mediates TGF-β-induced triple-negative breast cancer plasticity and chemoresistance
Source: Oncogene. 2021 Mar 5;40(14):2509–23. doi: 10.1038/s41388-021-01711-x (PMC8032554; doi:10.1038/s41388-021-01711-x)

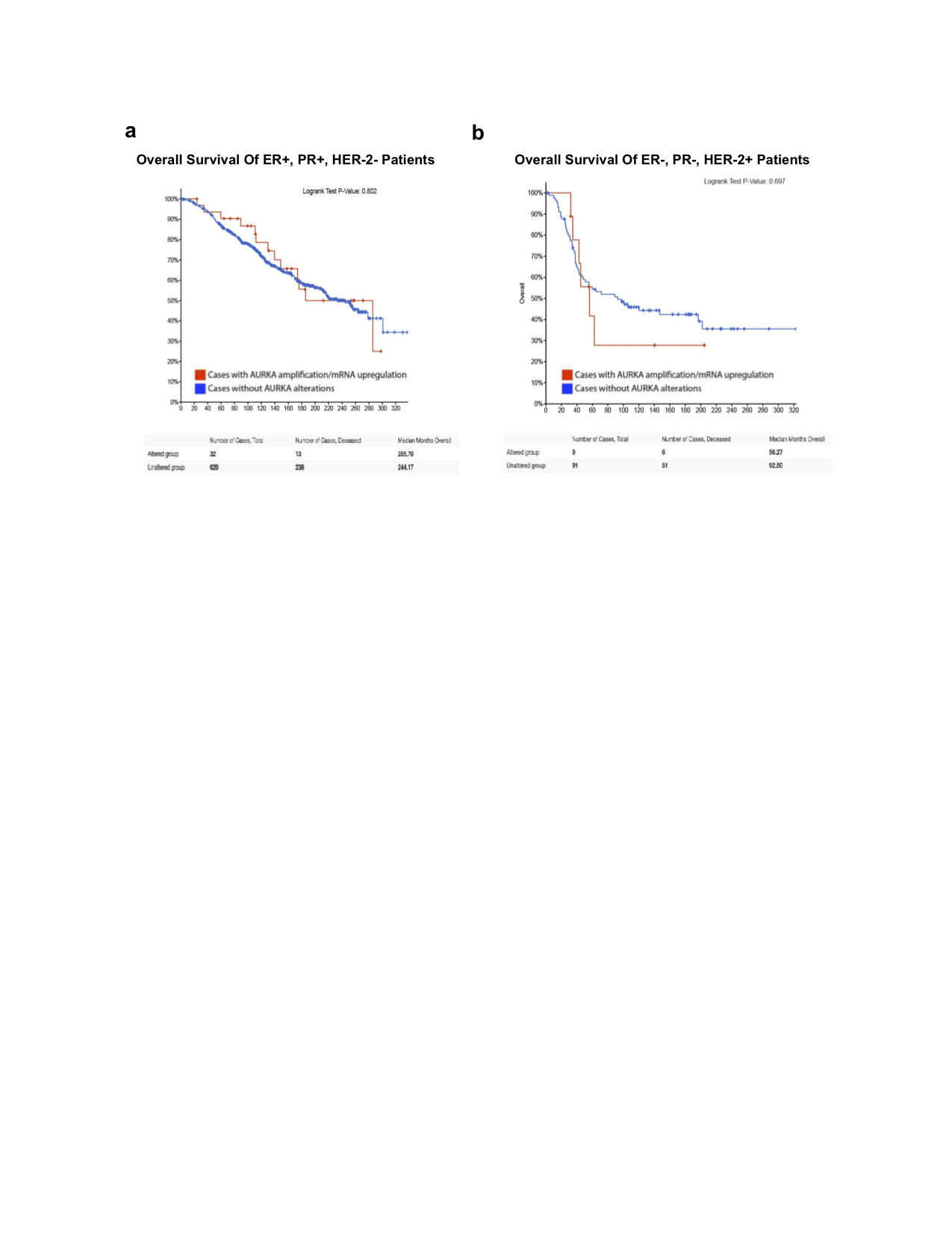

Supplement: Supplementary file 2 — SFIGURE 1 [file 41388_2021_1711_MOESM2_ESM.tif]

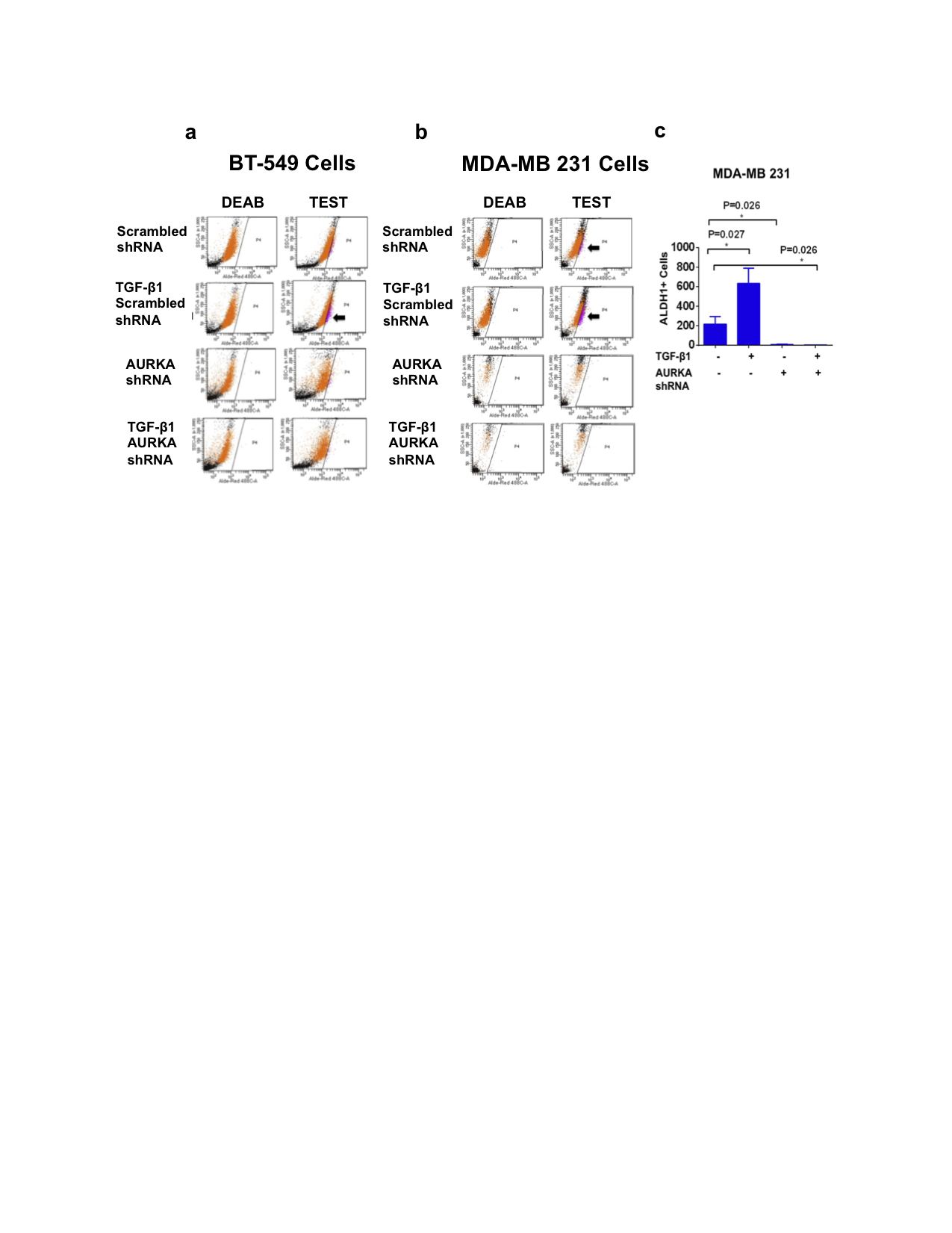

Supplement: Supplementary file 3 — SFIGURE 2 [file 41388_2021_1711_MOESM3_ESM.tif]

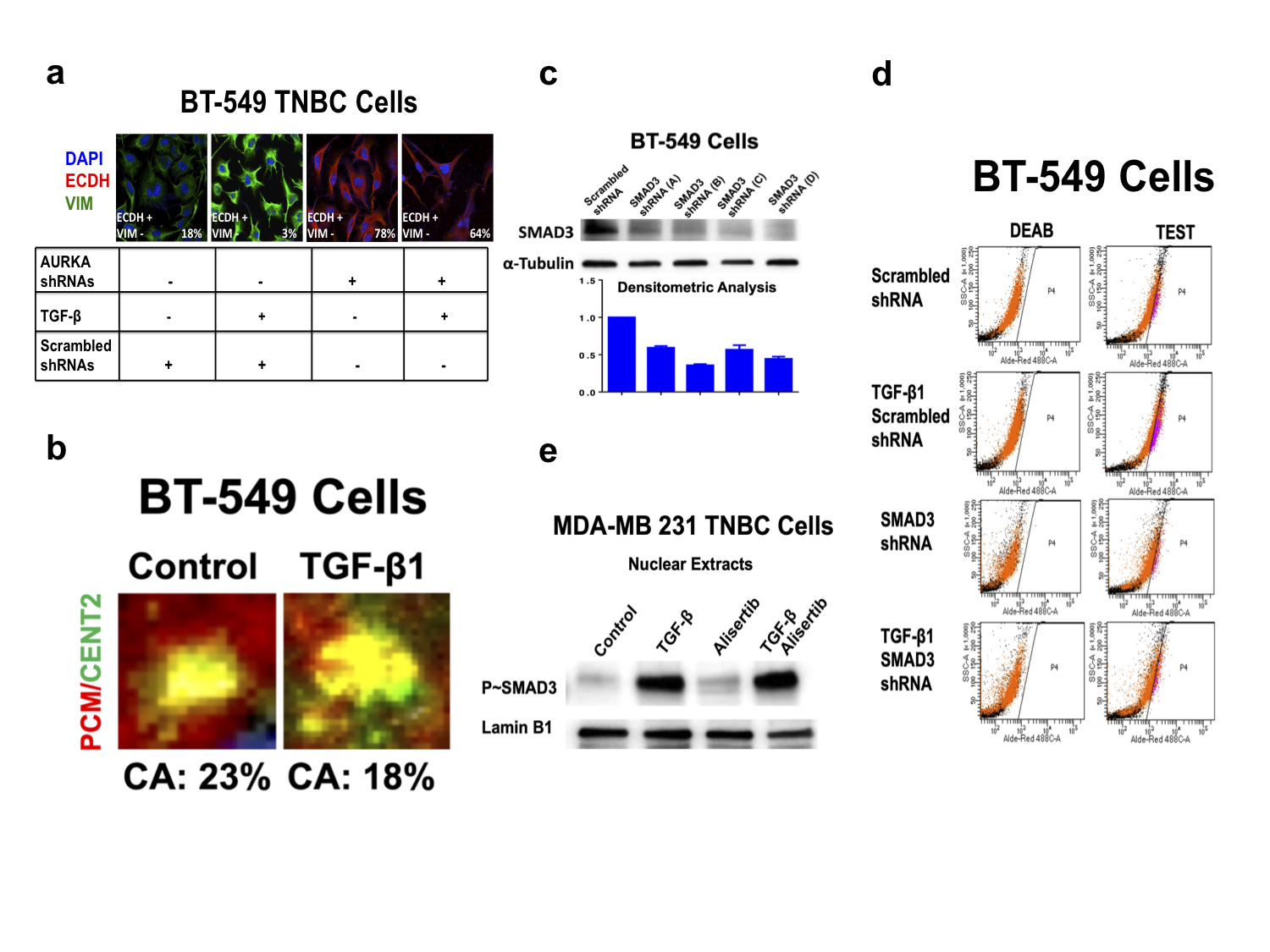

Supplement: Supplementary file 4 — SFIGURE 3 [file 41388_2021_1711_MOESM4_ESM.tif]

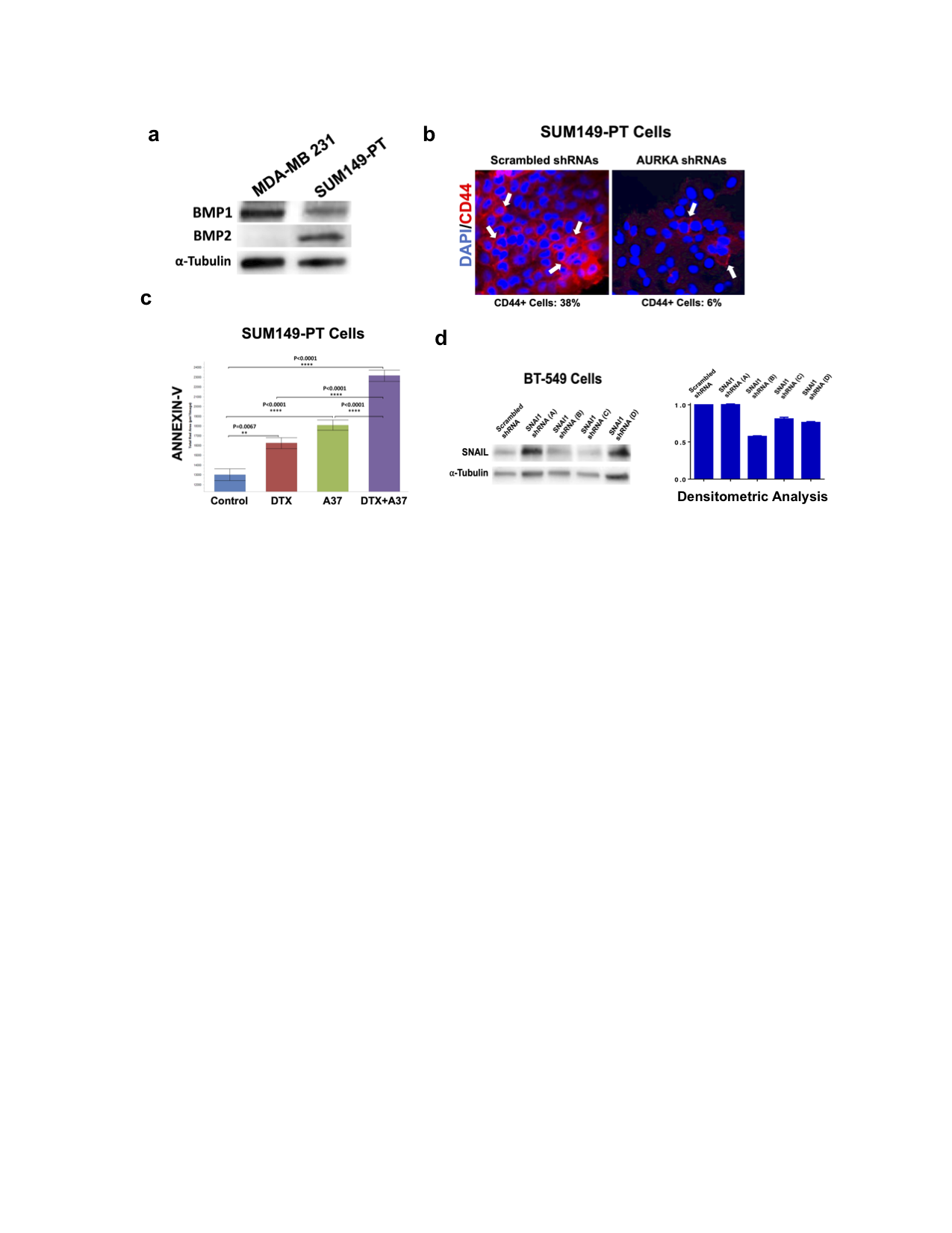

Supplement: Supplementary file 5 — SFIGURE 4 [file 41388_2021_1711_MOESM5_ESM.tif]

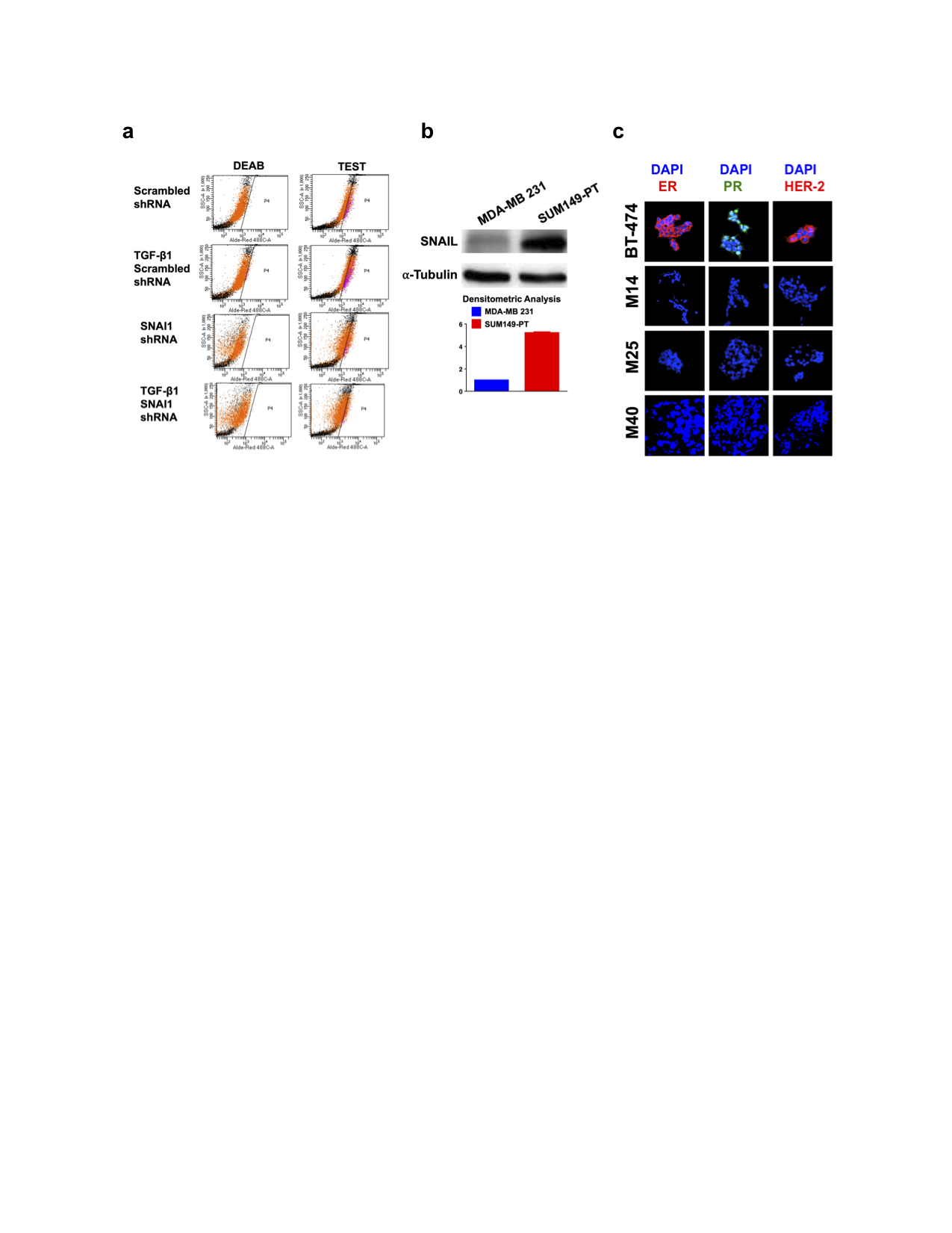

Supplement: Supplementary file 6 — SFIGURE 5 [file 41388_2021_1711_MOESM6_ESM.tif]

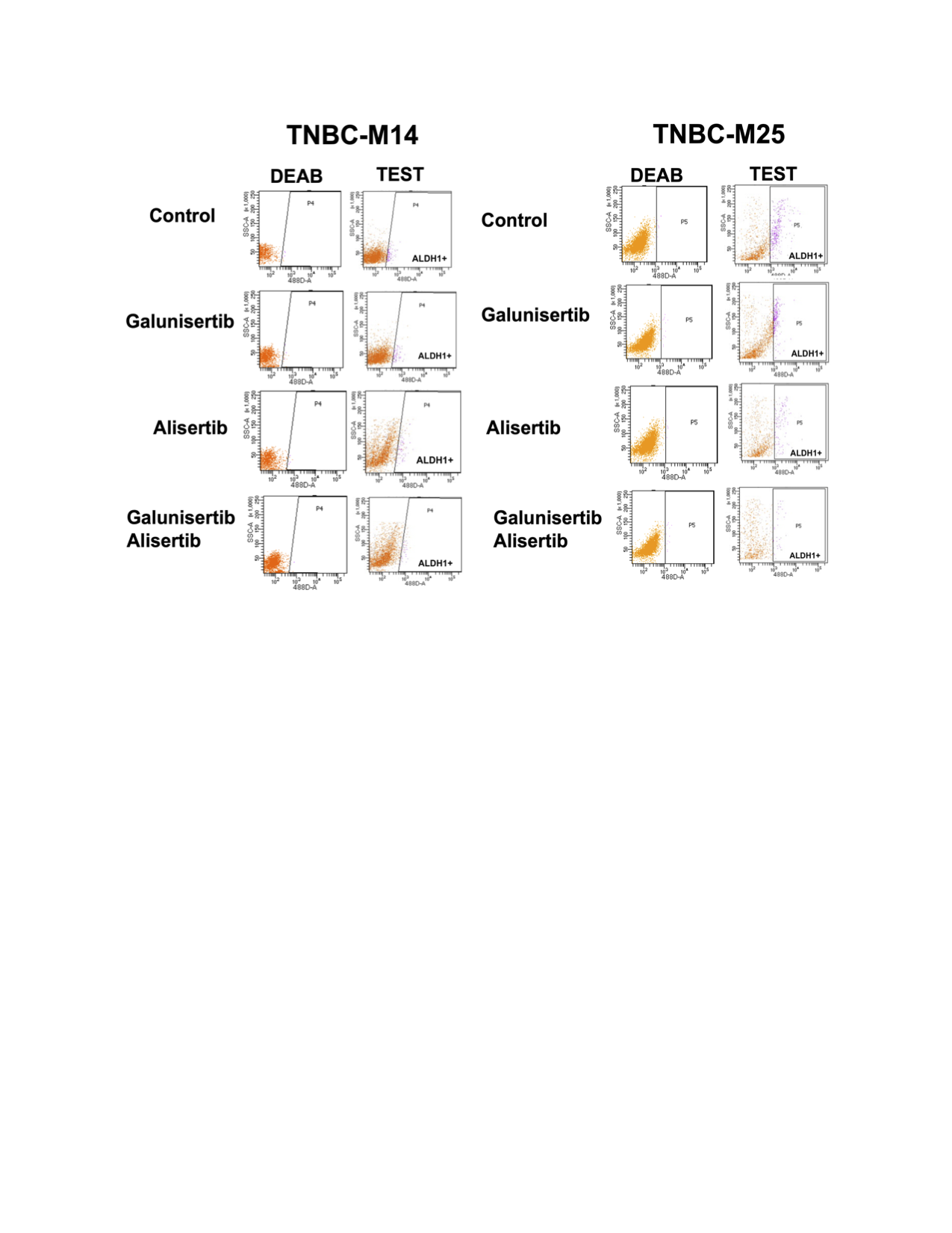

Supplement: Supplementary file 7 — SFIGURE 6 [file 41388_2021_1711_MOESM7_ESM.tif]

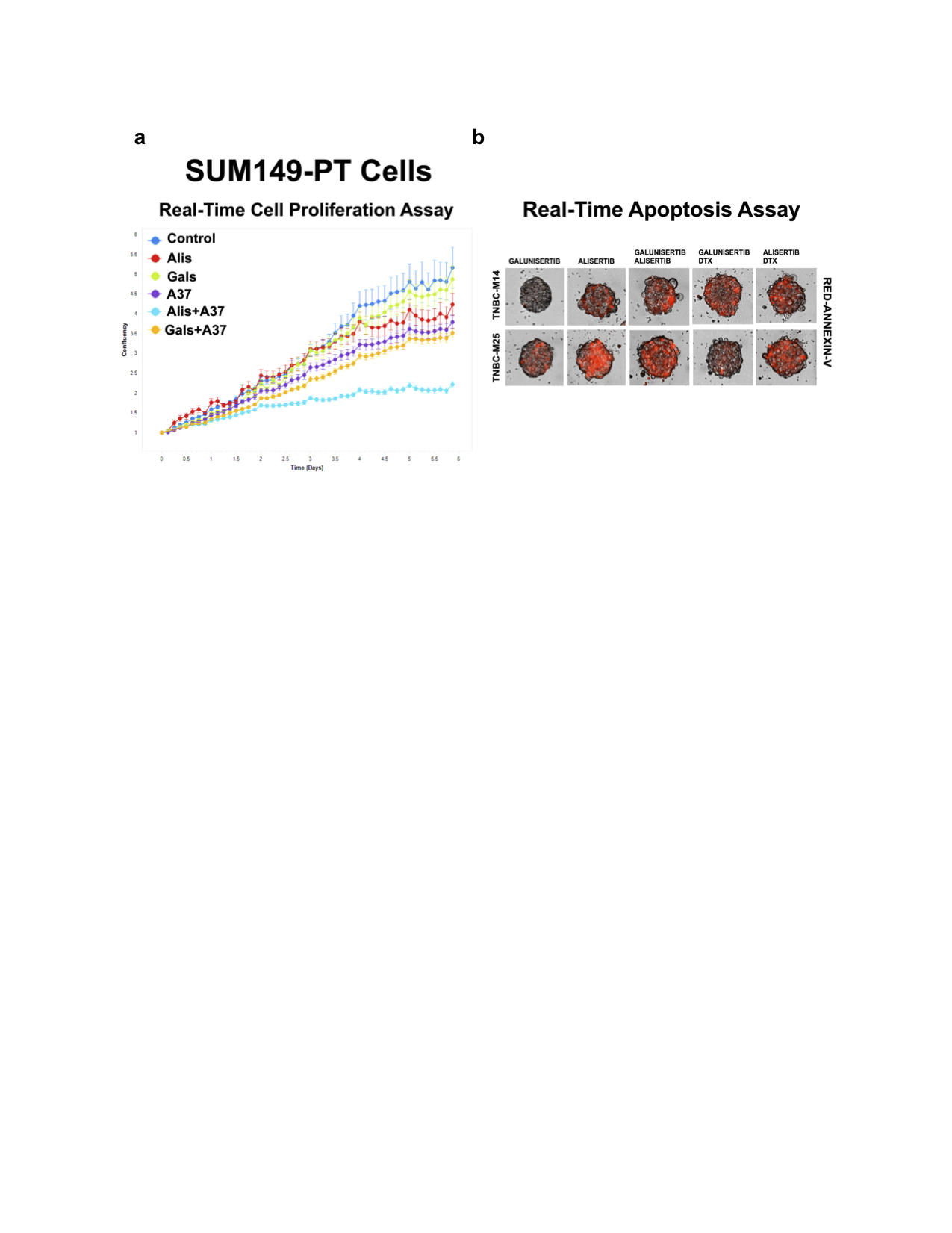

Supplement: Supplementary file 8 — SFIGURE 7 [file 41388_2021_1711_MOESM8_ESM.tif]
